# Alternative adjustment for seasonality and long-term time-trend in time-series analysis for long-term environmental exposures and disease counts

**DOI:** 10.1186/s12874-020-01199-1

**Published:** 2021-01-04

**Authors:** Honghyok Kim, Jong-Tae Lee, Kelvin C. Fong, Michelle L. Bell

**Affiliations:** 1 School of the Environment, Yale University, 195 Prospect Street, New Haven, CT 06511 USA; 2 BK21PLUS Program in ‘Embodiment: Health –Society Interaction’, Department of Public Health Science, Graduate School, Korea University, Seoul, Republic of Korea; 3 School of Health Policy and Management, College of Health Science, Korea University, Seoul, Republic of Korea

**Keywords:** Case-only data, Long-term exposure, Time-series analysis, Seasonality, Time-trend, Environmental exposure

## Abstract

**Background:**

Time-series analysis with case-only data is a prominent method for the effect of environmental determinants on disease events in environmental epidemiology. In this analysis, adjustment for seasonality and long-term time-trend is crucial to obtain valid findings. When applying this analysis for long-term exposure (e.g., months, years) of which effects are usually studied via survival analysis with individual-level longitudinal data, unlike its application for short-term exposure (e.g., days, weeks), a standard adjustment method for seasonality and long-term time-trend can extremely inflate standard error of coefficient estimates of the effects. Given that individual-level longitudinal data are difficult to construct and often available to limited populations, if this inflation of standard error can be solved, rich case-only data over regions and countries would be very useful to test a variety of research hypotheses considering unique local contexts.

**Methods:**

We discuss adjustment methods for seasonality and time-trend used in time-series analysis in environmental epidemiology and explain why standard errors can be inflated. We suggest alternative methods to solve this problem. We conduct simulation analyses based on real data for Seoul, South Korea, 2002–2013, and time-series analysis using real data for seven major South Korean cities, 2006–2013 to identify whether the association between long-term exposure and health outcomes can be estimated via time-series analysis with alternative adjustment methods.

**Results:**

Simulation analyses and real-data analysis confirmed that frequently used adjustment methods such as a spline function of a variable representing time extremely inflate standard errors of estimates for associations between long-term exposure and health outcomes. Instead, alternative methods such as a combination of functions of variables representing time can make sufficient adjustment with efficiency.

**Conclusions:**

Our findings suggest that time-series analysis with case-only data can be applied for estimating long-term exposure effects. Rich case-only data such as death certificates and hospitalization records combined with repeated measurements of environmental determinants across countries would have high potentials for investigating the effects of long-term exposure on health outcomes allowing for unique contexts of local populations.

**Supplementary Information:**

The online version contains supplementary material available at 10.1186/s12874-020-01199-1.

## Background

Time-series analysis is a prominent method to estimate the effect of short-term (e.g., days, weeks) environmental exposures (e.g., air pollution, extreme weather) on health outcomes [1, 2]. Time-series studies form the basis for our understanding of environmentally susceptible populations and the number of adverse health outcomes that can be potentially avoided if an adverse exposure level were to be reduced. Time-series data can consist of case-only records such as death certificates, hospitalization records, and insurance claims. So, time-series analysis with rich case-only data worldwide can contribute to not only the identification of the relationships between environmental exposures and a variety of health outcomes but also the improvement of generalizability of scientific findings, given that it is common that the health effects of environmental exposure are heterogeneous across regions and countries due to numerous modifiers such as demographics, socioeconomic positions and health behaviors [3–6]. For example, a recent study collected time-series data for mortality and air pollution over 652 cities across 24 countries [4] and found that the mortality effect of short-term exposure to air pollution may differ by countries.

In addition to short-term exposure effects, some environmental exposures, such as air pollution, have long-term (e.g., several months, years) exposure effects on health. These effects that are generally stronger than short-term exposure effects are usually investigated via cohort data. In cohort studies, exposure is estimated over long time periods, often accounting for mobility of study participants [1]. Cohort data with location information over time allow for more accurate assessment of exposure over long-time frames as participants are followed throughout time and changes in residence, which could affect exposure. However, cohort data are costly and difficult to construct, available for limited populations, and sometimes not representative for the general population so that generalizability for the magnitude of exposure effects is often limited: for example, most of the published air pollution cohort studies for Western countries, several for East Asian countries, and none for the rest of the world [6]. In contrast, registry data, such as death certificates and hospitalization records, are rich in many countries, but usually do not have information for mobility. This leads to higher potential exposure misclassification in studies of long-term exposure compared to studies of short-term exposure, which assume that participants had the same exposure for a few days prior to the event. Nevertheless, for general populations, such exposure misclassification is likely to result in underestimation [7, 8], which would be a basis of the conservative view if case-only data are used for inferring long-term exposure effects (i.e., understating the effects rather than overstating them).

A critical challenge for applying time-series analysis with case-only data into estimating the effect of long-term exposure on health outcomes is to adjust for seasonality and long-term time-trend. Unlike its application for short-term exposure, adjustment for seasonality and long-term time-trend may mask a real signal of the effect of long-term exposure on an health outcome [9, 10] and can greatly reduce temporal variability of residual time-series of long-term exposure that is necessary to detect the signal [11]. So, the adjustment can inflate standard error of estimates, which make it difficult to make an inference of the effect of interest. In this regard, time-series studies in environmental epidemiology have been generally focused on short-term exposure, usually of a few days, at most about 40–60 days of an exposure time-window [10, 12–17]. In this paper, we address issues of adjustment for seasonality and long-term time-trend in estimating the effects of long-term exposure on health outcomes.

In the following sections, we discuss adjustment methods frequently used in time-series studies and identify possible problems in estimating the effect of long-term exposure when such methods are used. To solve these problems, we suggest alterative adjustment methods. We present statistical simulations and real data analysis to demonstrate that the effects of long-term exposure can be estimated via time-series analysis with alternative adjustment methods.

## Methods

### Model formulation

Suppose that an individual’s hazard is estimated by a multiplicative hazard model with exposure variables, and a study population is a general population that can be seen as an open cohort. For shared environmental exposure over all individuals in a study population at time *t* and *X*_*t*_, population-averaged effect can be estimated using Poisson regression model with aggregated-level time-series data as follows [18]:

$$ \log \left(E\left[{Y}_t\right]\right)=\alpha +e\left({X}_t,\dots, {X}_{t-L}\right)+f\left({\boldsymbol{Z}}_t,\dots, {\boldsymbol{Z}}_{t-K}\right)+g(t) $$

where *Y* is the number of events and ***Z*** is a set of measured potential confounders such as temperature (for air pollution studies), relative humidity, and influenza epidemic. *e(X*_*t*_*,…,X*_*t-L*_*)* is a function of the population-averaged effect of *X* from lag0 (same day) to lag*L* (i.e., cumulative exposure for *L+ 1* days) on *Y*. In the distributed lag model framework [19], this function is generalized to ${\sum}_{l=0}^L{\beta}_l{X}_{t-l}$. The overall cumulative coefficient (i.e., the logarithm of the overall cumulative relative rate/risk) is $\overline{\beta}={\sum}_{l=0}^L{\beta}_l$. In practice, an observable *e(X*_*t*_*,…,X*_*t-L*_*)* may not be equal to *e(X*_*t*_*,…,X*_*t-L*_*)* due to mortality displacement and/or an exposure measurement error [20, 21]. *g(t)* is a function of the population-averaged effect of unmeasured risk factors that shape seasonality and time-trend in a time-series of *Y* [18]. Since *X* has seasonality and time-trend, confounding could arise. To control for this confounding, several techniques have been used in the time-series literature. We discuss them in the context of estimating the effect of long-term *X* (i.e., *X*_*t*_*,…,X*_*t-L*_, *L*>months or years) on *Y*.

### Adjustment for seasonality and long-term time-trend

#### Detrending prior to regression

Prior to a regression analysis, seasonality and time-trend in an exposure series and an outcome series can be decomposed or removed by algorithms [14, 22, 23]. Detrended time-series can be used to estimate the effect of short-term *X* on *Y*.

For estimating the effect of long-term *X* on *Y*, detrending prior to regression is more challenging because a real signal of the effect of long-term *X* on *Y* may also be removed. For example, detrending a time-series of *Y* will remove some long-term fluctuation of *Y* that is formed by ${\sum}_{l=0}^L{\beta}_l{X}_{t-l}$. Consequently, an estimate of the effect of long-term *X* on *Y* would be biased toward the null.

#### Adjustment in regression models

Many time-series studies in environmental epidemiology adjust for seasonality and long-term time-trend by adding a variable or a set of variables representing time (*t*) into regression models [1, 2, 11, 24]. Natural cubic splines (NCS) with some degrees of freedom (df) per year are frequently used, and its performance for adjustment was tested by simulation studies [24–26]. We use NCS(*t*,*p*df/year) to denote this in generalized linear models where *t* represents a variable representing time and *p* reflects the degrees of freedom. Non-parametric splines in generalized additive models are also frequently used, but for simplicity, we focus on the former as they may provide less biased estimates [11, 24]. In mortality studies, 3–8df/year is often used [24, 26, 27], but the optimal df may decrease depending on whether other variables explain seasonality to some extent (e.g., influenza epidemic, heat wave) are included in models [27].

Often, a combination of some variables is used [28, 29], such as NCS of calendar time of the year called *day of the year* (or *day of the season*) and an indicator of *year* (i.e., dummy variables) or equivalent (e.g., NCS of *year*). We use NCS(doy,*p*df)+I(year) to denote NCS of day of the year (doy) and an indicator of year. But, NCS(doy,*p*df) may not allow for seasonality that may vary between years. To address this, we additionally consider an NCS of the order of *week* throughout a study period, NCS(week,*q*df) or an NCS of the order of *month* throughout a study period NCS(month,*r*df). NCS(doy,*p*df)+I(year)+NCS(week,*q*df)+NCS(month,*r*df) can collectively capture seasonality and long-term time-trend.

**Fig. 1:**
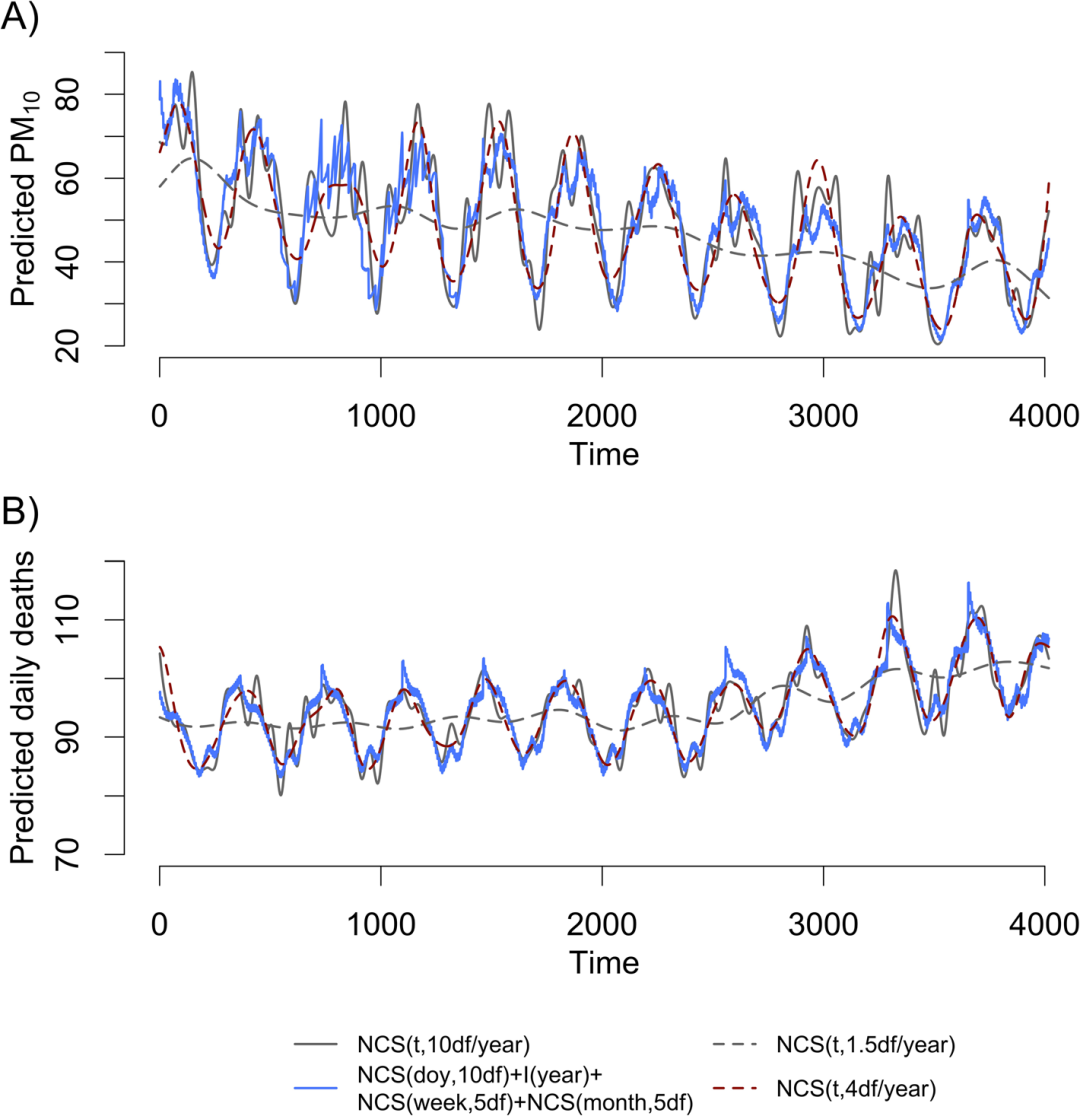
Seasonality and time-trend in daily time-series in Seoul, 2002–2013. **a** PM_10_ concentration (μg/m^3^). **b** all-cause mortality (number of deaths/day), estimated by different functions of variables representing time. Abbreviation: df, degrees of freedom; doy, day of the week; NCS, natural cubic spline; I(year), an indicator function of year throughout the study period; NCS(doy,10df), NCS of day of the year with 10df; NCS(month,5df), NCS of the order of month throughout the study period with 5df; NCS(t,*p*df/year), NCS of time throughout the study period with *p*df per year; NCS(week,5df), NCS of the order of week throughout the study period with 5df; PM_10_, particulate matter with aerodynamic diameter ≤10 μm

To present comparison between these functions, we smoothed daily time-series of PM_10_ and mortality in Seoul, South Korea, 2002–2013 through NCS with various degrees of freedoms (Fig. 1). Hourly measurement of PM_10_ concentration and all-cause mortality (International Classification of Diseases-10th, A00-R99) were obtained from the National Institute of Environmental Research and Statistics Korea, respectively, and hourly measurements from multiple monitors within Seoul on a given day were averaged. Figure 1 shows that NCS(*t*,4df/year) can capture seasonality and long-term time-trend. NCS(*t*,10df/year) can further capture fluctuations more aggressively, which in cases of all-cause mortality, may be related to other environmental exposures such as heat waves [27]. NCS(doy,10df)+I(year)+ NCS(week,5df)+NCS(month,5df) also captures seasonality and long-term time-trend although there are some differences compared to NCS(*t*,10df/year): for example, a peak around 3300 time point (i.e., day). Such differences may be captured using additional dummy variables.

In estimating the effect of long-term *X* on *Y*, standard errors can become very high, so that unstable estimates or spuriously significant estimates can come out. This is often referred to as overfitting as analogous of collinearity problems. To illustrate overfitting, we regressed a one-year moving average of PM_10_ concentration in Seoul, 2002–2013 on functions of variables representing time. Table 1 presents variance of residuals of this moving average and *R*^*2*^. NCS(*t*,4df/year) explains nearly all variability of the moving average (*R*^*2*^=99.3%). In comparison to NCS(*t*,4df/year) and NCS(*t*,10df/year), NCS(doy,10df)+I(year) and NCS(doy,10df)+I(year) +NCS(week,5df)+NCS(month,5df) explains to a lesser extent variability of the moving average (*R*^*2*^=96.6 and 97.1% respectively). The perhaps seemingly small difference in residual variances between these methods could make striking difference in standard errors. Assuming that there are no other covariates to be considered, standard errors may be approximately 17 times higher when NCS(*t*,4df/year) is used than when NCS(doy,10df)+I(year) +NCS(week,5df)+NCS(month,5df) is used, which is based on a ratio of the two standard deviations of the residuals [30].

**Table 1:** Residual variance of one-year moving average of PM_10_ concentration in Seoul, South Korea, 2002–2013

Adjustment method for seasonality and long-term time-trend	Residual variance	***R***^***2***^ (%)
Not adjusted	71.080	
NCS(*t*,10df/year)	0.185	99.7
NCS(*t*,4df/year)	0.505	99.3
NCS(*t*,1.5df/year)	1.029	98.6
NCS(doy,10df)+I(year)+NCS(week,20df)+NCS(month,20df)	0.675	99.1
NCS(doy,10df)+I(year)+NCS(week,5df)+NCS(month,5df)	2.088	97.1
NCS(doy,10df)+I(year)	2.417	96.6

Abbreviations: *df* degrees of freedom, *doy* day of the year, *I(year)* an indicator function of year throughout the study period, *NCS* natural cubic spline, *NCS(doy,pdf)* NCS of doy with *p*df, *NCS(month,pdf)* NCS of the order of month throughout the study period with *p*df, *NCS(t,pdf/year)* NCS of time throughout the study period with *p*df per year, *NCS(week,pdf)* NCS of the order of week throughout the study period with *p*df, *PM*_*10*_ particulate matter with aerodynamic diameter ≤10 μm

To maintain variability in residuals of long-term *X* using NCS(*t*,*p*df/year), df needs to be reduced, but low df results in loss of adjustment for seasonality. For example, NCS(*t*,1.5df/year) does not adequately capture seasonality (Fig. 1b). Note that this function still captures variability of a one-year moving average of PM_10_ to a greater extent than NCS(doy,10df)+I(year)+NCS(week, 5df)+NCS(month, 5df) (Table 1). Thus, NCS(*t*,*p*df/year) may not be relevant for estimating the effect of long-term *X* on *Y*.

In summary, adjustment for seasonality and long-term time-trend should be made with avoiding reducing variability of *X* to a great extent. NCS(*t*,*p*df/year) as a standard method in time-series studies appears inefficient. A candidate we explored is a combination of some variables such as NCS(doy, *p*df)+I(year)+NCS(week,*q*df)+NCS(month,*r*df) with moderate df. Simulations and real-data analyses were conducted to test this method.

### Confounding by long-term association

Although our primary interest is to estimate the effect of long-term *X* on *Y*, whether the effect of short-term *X* on *Y* (e.g., ${\sum}_{l=0}^M{\beta}_l$ (*M*<few days or weeks) can be confounded by long-term association (e.g., ${\sum}_{l=M}^L{\beta}_l{X}_{t-l}$) merits investigation in that neighboring lagged variables can have an confounding effect [31] and long-term association can be seen as an weighted moving average of *X* that would contribute to seasonality and long-term time-trend of *Y*. If conventional adjustment for seasonality and a time-trend does not sufficiently adjust for long-term association, an inclusion of exposure variables may be needed. Simulations and real-data analyses were also conducted to explore confounding by long-term association.

### Simulation methods

We investigate performance of different functions to adjust for seasonality and long-term time-trend in estimating the effect of long-term *X* on *Y* using simulation analyses.

#### Generating simulation samples

**Fig. 2:**
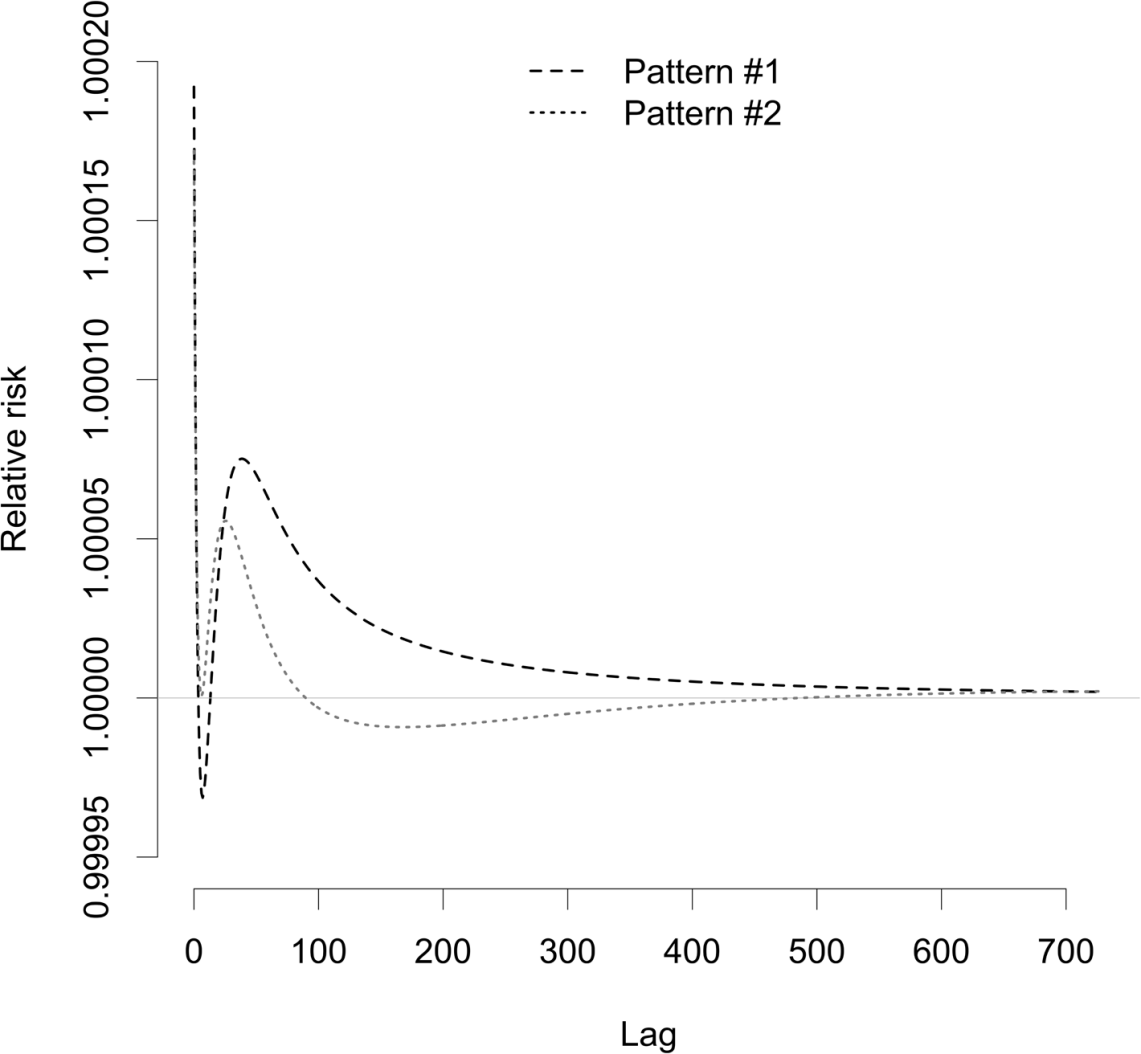
Two hypothetical observable lag patterns of the association between 1 μg/m^3^ increase of exposure to PM_10_ and mortality, used to generate simulation samples. Abbreviation: PM_10_, particulate matter with aerodynamic diameter ≤10 μm

Figure 2 shows two hypothetical observable lag patterns to generate simulation samples. The assumed overall cumulative coefficients were 0.01 and 0.001 per a 1 μg/m^3^ increase of PM_10_, for the observable lag patterns in Fig. 2a and b, respectively. We considered the effect of cumulative exposure on an outcome and mortality displacement; we did not consider exposure measurement errors. For example, epidemiologic evidence shows that the logarithm of the relative risk of the exposure to PM_2.5_ on mortality generally increases with the exposure duration (i.e., *L* increases in the distributed lag model framework) while the highest marginal increase arises at the most proximal time of the exposure [32, 33]. We considered two distributions of decreases in mortality due to mortality displacement (Fig. S1 in Additional file 1). Findings of many time-series studies focused on short-term exposure to PM imply that observable lag patterns for short-term exposure to air pollutants and mortality are non-linear [10, 12, 13, 15–17, 34] suggesting that short-term mortality displacement may play a role in shaping observable lag patterns.

We generated simulation samples for daily time-series of PM_10_ and mortality based on real data of Seoul, 2002–2013. For this, we regressed the logarithm of daily average of PM_10_ and the daily number of all-cause mortality cases (except for accidental causes) on functions of variables representing time and potential confounders in time-series studies, including a lag-structure of temperature from lag0–21, relative humidity, influenza epidemic, national holidays, and day of the week. For temperature and relative humidity, hourly measurements at the center of Seoul obtained from the Korean Meteorological Administration were averaged to daily values. Influenza epidemic was based on the number of hospital visits for influenza, which was obtained from the National Health Insurance System. To generate a time-series of PM_10_, values were sampled from normal distributions and then were exponentiated. A mean of normal distributions was predicted with values obtained from the regression model for the logarithm of PM_10_. Their standard deviation was estimated from the model (low concurvitiy) or the value divided by 10 (high concurvity) [24]. To generate time-series of all-cause mortality, the number of events were sampled from Poisson distributions. A mean of Poisson distributions was the sum of predicted values obtained from the Poisson regression model for all-cause mortality and a product of a lag pattern and distributed lags of generated PM_10_ series. Technical detail for this section is presented in Additional file 1. Fig. S2 in Additional file 1 shows an example of simulated sample of time-series.

#### Testing models

To adjust for seasonality and long-term time-trend in generated time-series samples, we used NCS(*t*,4df/year), NCS(*t*,10df/year), and NCS(doy,10df)+I(year)+NCS(week,5df)+NCS(month, 5df). We used constrained distributed lags of PM_10_ (lag0–730) to estimate the association between PM_10_ and mortality. We also used only two-day moving average of PM_10_ (lag0–1) to see whether the cumulative exposure to PM_10_ and mortality displacement (lag2–730) can have a confounding effect. We adjusted for a lag-structure of temperature, relative humidity, influenza epidemic, national holidays, and day of the week. Technical detail for this section is presented in Additional file 1.

### Methods for real data analysis

We conducted a two-stage time-series analysis to estimate the effect of long-term exposure to PM_10_ on mortality in seven major cities (Seoul, Busan, Daegu, Incheon, Gwangju, Daejeon, and Ulsan) of South Korea from 2006 to 2013. Datasets are described in Additional file 2. In the first stage, we fitted city-specific Poisson regression models with overdispersion considered. Constrained distributed lags of PM_10_ (lag0–365) were used as an exposure metric. A lag-structure of temperature (lag0–14 or lag0–21), O_3_ (lag0–45), relative humidity, and influenza epidemic was adjusted. Seasonality and long-term time-trend were also adjusted using (a) NCS(*t*,*p*df/year), (b) NCS(doy,*p*df)+I(year), or (c) NCS(doy,*p*df)+I(year)+NCS(week,*p*df)+ NCS(month,*p*df). Technical detail for modelling is provided in Additional file 2. In the second stage, we applied multivariate meta-analysis to pool city-specific estimates [35]. We compared pooled estimates of the association between adjustments for seasonality and long-term time-trend. This procedure was to check possible overfitting.

As sensitivity analyses, we used different dfs in each NCS. We also additionally adjusted for multiple dummy variables indicating days when deviations of seasonality were identified. For this additional adjustment, we found that days when predicted daily deaths of a city-specific model with NCS(doy,20df)+I(year)+NCS(week,3df)+NCS(month,3df) and those of a city-specific model with NCS(*t*,10df/year) did not coincide one another; if deviations from normal seasonal patterns exist across years, these two models would yield different predicted daily deaths because NCS(*t*,10df/year) is likely to capture deviations better. We used cut-off values to determine deviations of seasonality: |*A*_*t*_-*B*_*t*_|>a cut-off value where *A*_*t*_ is predicted logarithm of daily deaths from one model and *B*_*t*_ is predicted logarithm of daily deaths from the other model at time *t*. We varied a cut-off value from 98th percentile, 96th percentile, …, to 80th percentile of |*A*_*t*_-*B*_*t*_|.

### Software

We used R software 3.5.3 (R Foundation for Statistical Computing) for simulations and real-data analyses. We used *splines* package for NCS [36], *dlnm* package for distributed lags of PM_10_ and distributed lag non-linear terms of temperature [37], and *mvmeta* package for pooling city-specific estimates [35]. R codes for simulations and real-data analysis are provided in the first author’s website, http://hkimresearch.com, or Github, https://github.com/HonghyokKim/AlternativeAdjustment.

## Results

### Results of simulations

**Fig. 3:**
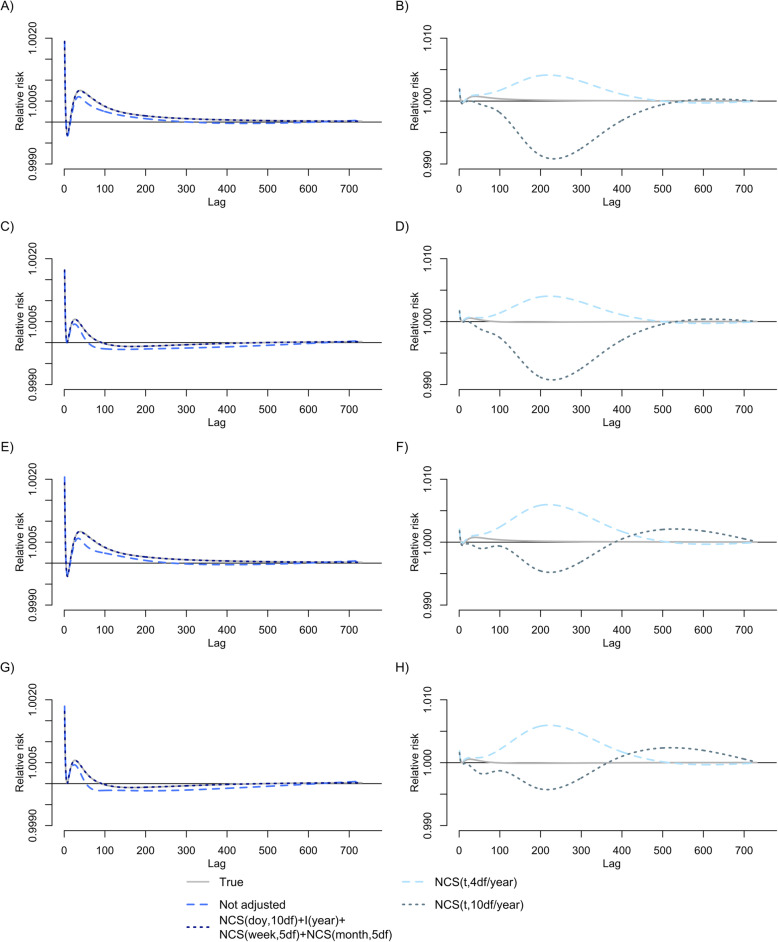
Estimated lag patterns of the association between PM_10_ and all-cause mortality by different methods to adjust for seasonality and time-trend over 5000 samples of different simulation settings based on time-series data for Seoul, 2002–2013. **a** and **b** Low concurvity and Observable lag pattern 1. **c** and **d** Low concurvity and Observable lag pattern 2. **e** and **f** High concurvity and Observable lag pattern 1. **g** and **h** High concurvity and Observable lag pattern 2. Abbreviation: df, degrees of freedom; doy, day of the week; I(year), an indicator function of year throughout the study period; NCS, natural cubic spline; NCS(doy,10df), NCS of day of the year with 10df; NCS(month,5df), NCS of the order of month throughout the study period with 5df; NCS(t,10df/year), NCS of time throughout the study period with 10df per year; NCS(week,5df), NCS of the order of week throughout the study period with 5df; PM_10_, particulate matter with aerodynamic diameter ≤10 μm

Table 2 presents bias, standard deviation, and nominal coverage of 95% confidence interval for the overall cumulative association between distributed lags of PM_10_ (lag0–730) and mortality. Models adjusted for NCS(doy,10df)+I(year)+NCS(week,5df)+NCS(month,5df) produced estimates of the overall cumulative coefficient with negligible bias and almost perfect coverage of 95% confidence intervals (Table 2). Lag patterns were estimated accurately (Fig. 3). As expected by very small residual variance of PM_10_ as in Table 1, models adjusted for NCS(t, 4df/year) and NCS(t,10df/year) produced very large standard errors, indicating overfitting. These models also produced lag-mortality associations that deviated far from lag patterns used to generate simulation samples (Fig. 3).

**Table 2:** Bias, standard deviation, nominal coverage of 95% confidence interval for the overall cumulative association between distributed lags of PM_10_ (lag0–730) and all-cause mortality estimated by a model with different adjustment methods for seasonality and long-term time-trend over 10,000 samples of different simulation settings based on time-series data for Seoul, 2002–2013

Concurvity^a^	Adjustment^b^	Observable Lag Pattern 1^a^			Observable Lag Pattern 2^a^		
		Bias (%)	SD^c^	Coverage^d^(%)	Bias (%)	SD^c^	Coverage^d^(%)
Low	Not adjusted	−50.1	0.004	0	− 509.2	0.004	0
	NCS(*t*,4df/year)	885.2	1.457	89.6	9259.9	1.459	88.9
	NCS(*t*,10df/year)	− 2239.6	19.994	94.3	−21,974.9	19.651	94.7
	10,5,5	−0.5	0.047	95.0	−2.6	0.047	94.9
High	Not adjusted	−54.1	0.003	0	− 549.4	0.003	0
	NCS(*t*,4df/year)	1346.0	1.828	88.7	14,086.5	1.825	87.9
	NCS(*t*,10df/year)	− 496.0	24.822	94.9	− 2649.2	24.971	94.6
	10,5,5	−0.4	0.056	94.8	2.9	0.055	95.0

Abbreviations: *df* degrees of freedom, *NCS* natural cubic spline, *NCS(t,pdf/year)* NCS of time throughout the study period with *p*df per year, *PM*_*10*_ particulate matter with aerodynamic diameter ≤10 μm, *SD* standard deviation

^a^Simulation settings: Low concurvity & Observable lag pattern 1, Low concurvity & Observable lag pattern 2, High concurvity & Observable lag pattern 1, and High concurvity & Observable lag pattern 2; Observable lag patterns 1–2 are provided in Fig. 2

^b^NCS(doy,10df)+I(year)+NCS(week,5df)+NCS(month,5df) is described as 10,5,5; NCS(doy,10df) is NCS of day of the year with 10df; I(year) is an indicator function of year throughout the study period; NCS(week,5df) is NCS of the order of week throughout the study period with 5df; NCS(month,5df) is NCS of the order of month throughout the study period with 5df

^c^SD of estimates of the overall cumulative coefficient (as 10 μg/m^3^ increase of PM_10_)

^d^Nominal coverage of 95% confidence intervals

Our simulation results suggest that the effect of short-term exposure to PM_10_ on all-cause mortality may be confounded by the effect of long-term exposure to PM_10_ on all-cause mortality in some circumstances. For example, for observable lag pattern #2, bias with respect to a moving average of PM_10_ from lag0–1 in its relation with all-cause mortality was − 11.3% when NCS(*t*,10df/year) was used and PM_10_ from lag2–730 was not adjusted (Table S1 in Additional file 1). When PM_10_ from lag2–730 was adjusted, the bias decreased to 2.8%. The bias decreased with higher df. For observable lag pattern #1, the bias decreased to 0.4% from 4df to 10df when PM_10_ from lag2–730 was not adjusted.

### Results of real-data analyses

Table 3 presents percentage increases in all-cause mortality risk per a 10 μg/m^3^ increase in two-day moving average of PM_10_ (lag0–1) or PM_10_ for one-year (i.e., lag0–365) in seven major cities of South Korea. When PM_10_ (lag2–365) was adjusted, the percentage increases in all-cause mortality were 0.14% (95% confidence interval: − 0.06, 0.34) for NCS(t,10df/year) and 0.14% (− 0.03, 0.31) for NCS(doy, 20df)+I(year)+NCS(week,3df)+NCS(month,3df).

**Table 3:** Percentage increase and 95% confidence intervals of all-cause mortality risk per a 10 μg/m^3^ Increase in two-day moving average of PM_10_ (lag0–1) or one-year distributed lags of PM_10_ (lag0–365) in seven major cities of South Korea, 2006–2013

Adjustment method for seasonality and long-term time-trend^a^	Two-day moving average (lag0–1)				One-year distributed lags	
	lag2–365 not adjusted		lag2–365 adjusted		(lag0–365)	
	PI	95% CI	PI	95% CI	PI	95% CI
Not adjusted	−0.44	−0.71, − 0.16	0.08	−0.08, 0.24	−9.46	−13.27, − 5.48
NCS(t,7df/year)	0.16	0, 0.31	0.12	−0.06, 0.31	4.67	−51.66, 126.62
NCS(t,10df/year)	0.15	−0.01, 0.31	0.14	−0.06, 0.34	−20.60	−76.83, 172.13
NCS(t,12df/year)	0.15	−0.01, 0.31	0.20	−0.01, 0.40	−35.64	−84.39, 165.33
NCS(doy, 20df)+I(year)	0.13	−0.03, 0.28	0.17	0, 0.33	2.86	−2.13, 8.10
10,3,3	0.10	−0.05, 0.26	0.14	−0.05, 0.34	4.40	−0.10, 9.11
20,3,3	0.12	−0.04, 0.27	0.14	−0.03, 0.31	4.66	0.14, 9.38
30,3,3	0.12	−0.04, 0.28	0.15	−0.02, 0.32	4.64	0.07, 9.41
20,4,4	0.12	−0.03, 0.28	0.14	−0.03, 0.31	5.54	1.32, 9.95
20,5,5	0.11	−0.04, 0.27	0.12	−0.05, 0.29	4.55	−0.93, 10.34
20,6,6	0.08	−0.08, 0.24	0.09	−0.09, 0.28	0.50	−5.75, 7.18
20,10,10	0.08	−0.08, 0.23	0.12	−0.09, 0.34	5.45	−4.81, 16.83
20,15,15	0.09	−0.07, 0.25	0.13	−0.07, 0.34	−4.72	−22.75, 17.51

Abbreviations: *CI* confidence interval, *df* degrees of freedom, *doy* day of the year, *I(year)* an indicator function of year throughout the study period, *NCS* natural cubic spline, *NCS(doy,pdf)* NCS of doy with *p*df, *NCS(t,pdf/year)* NCS of time throughout the study period with *p*df per year, *PI* percentage increase, *PM*_*10*_ particulate matter with aerodynamic diameter ≤10 μm

^a^NCS(doy,*p*df)+I(year)+NCS(week,*q*df)+NCS(month,*r*df) is described as *p*,*q*,*r*; NCS(week,*q*df) is NCS of the order of week throughout the study period with *q*df; NCS(month,*r*df) is NCS of the order of month throughout the study period with *r*df

The percentage increase in all-cause mortality risk for a 10 μg/m^3^ increase of PM_10_ for 1 year (i.e., lag0–365) was 4.66% (0.14, 9.38) when NCS(doy,20df)+I(year)+NCS(week,3df)+ NCS(month,3df) was used (Table 3). This percentage increase was stable when different df were used for each NCS unless df were too high for NCS(week,*q*df) and NCS(month,*r*df). For example, estimates were variable with very wide confidence intervals for 6, 10, and 15 df for each NCS (Table 3). When NCS(*t*,*p*df/year) was used, estimates across different df were extremely variable with very wide confidence intervals, indicating overfitting (Table 3). For cardiovascular mortality and respiratory mortality, we found similar patterns of estimates as those found for all-cause mortality. Percentage increase in cardiovascular mortality risk and respiratory mortality risk for a 10 μg/m^3^ increase of PM_10_ for 1 year was 10.76% (0.62, 21.93) and 9.34% (− 17.77, 45.40), respectively (Tables S2–3 in Additional file 2).

Figure S3 in Additional file 2 presents an example of differences between time-series of predicted logarithm of daily deaths from city-specific models with NCS(doy,20df)+I(year)+NCS(week,3df)+ NCS(month, 3df) and city-specific models with NCS(*t*,10df/year). Since a lag-structure of temperature, air pollutants, and other variables such as influenza epidemics were all used, both models predicted daily mortality series similarly. Nevertheless, some deviations of seasonality were also identified: for example, about 1000–1250 time points (i.e., days) and around 1800 time point in Seoul (Fig. S3A). We adjusted for such deviations using multiple dummy variables and found our results robust to these additional adjustments (Fig. S4 in Additional file 2).

**Fig. 4:**
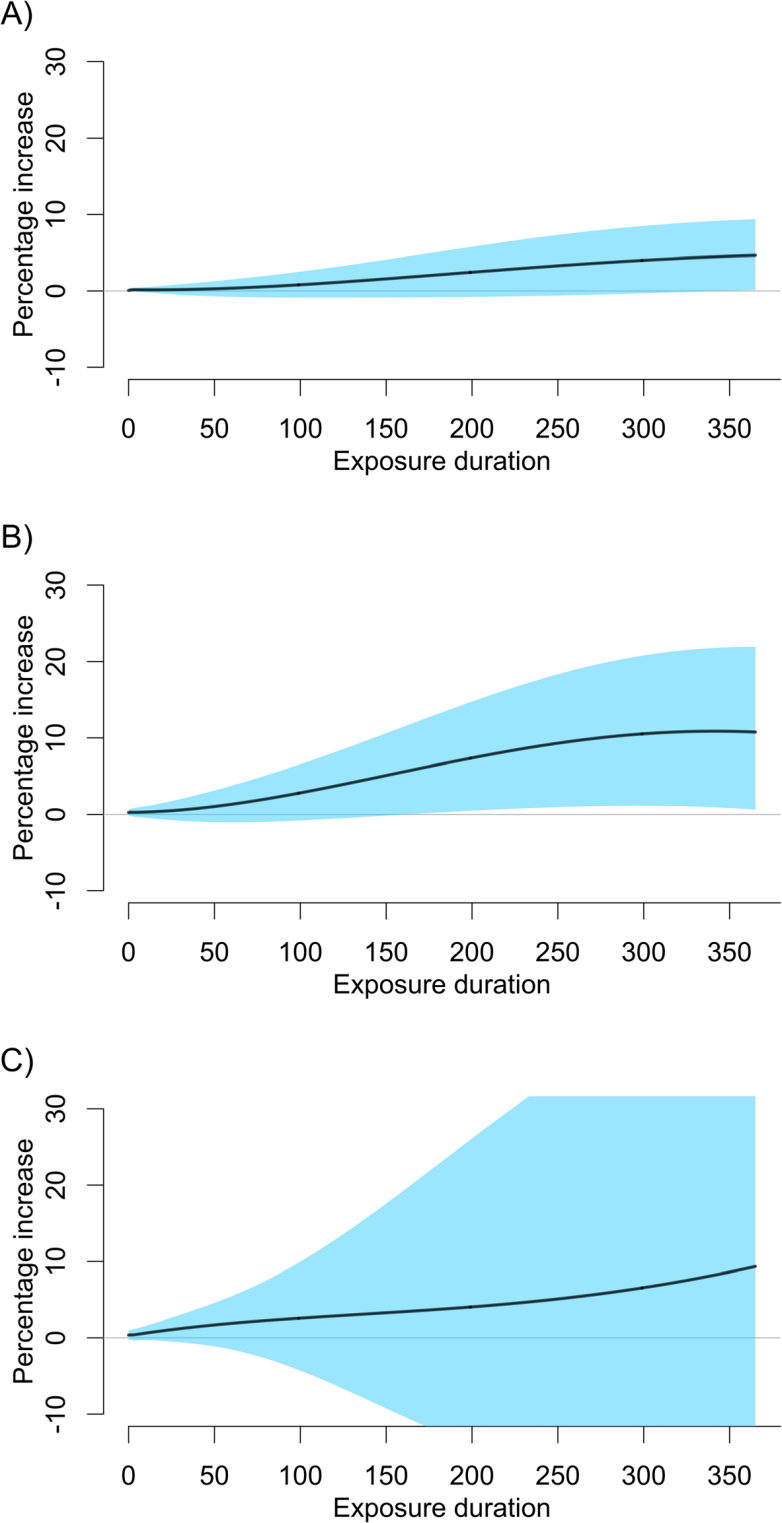
Percentage increase of mortality risk per a 10 μg/m^3^ increase of PM_10_ by exposure duration in seven major cities of South Korea, 2006–2013. **a** All-cause mortality. **b** Cardiovascular mortality. **c** Respiratory mortality

**Fig. 5:**
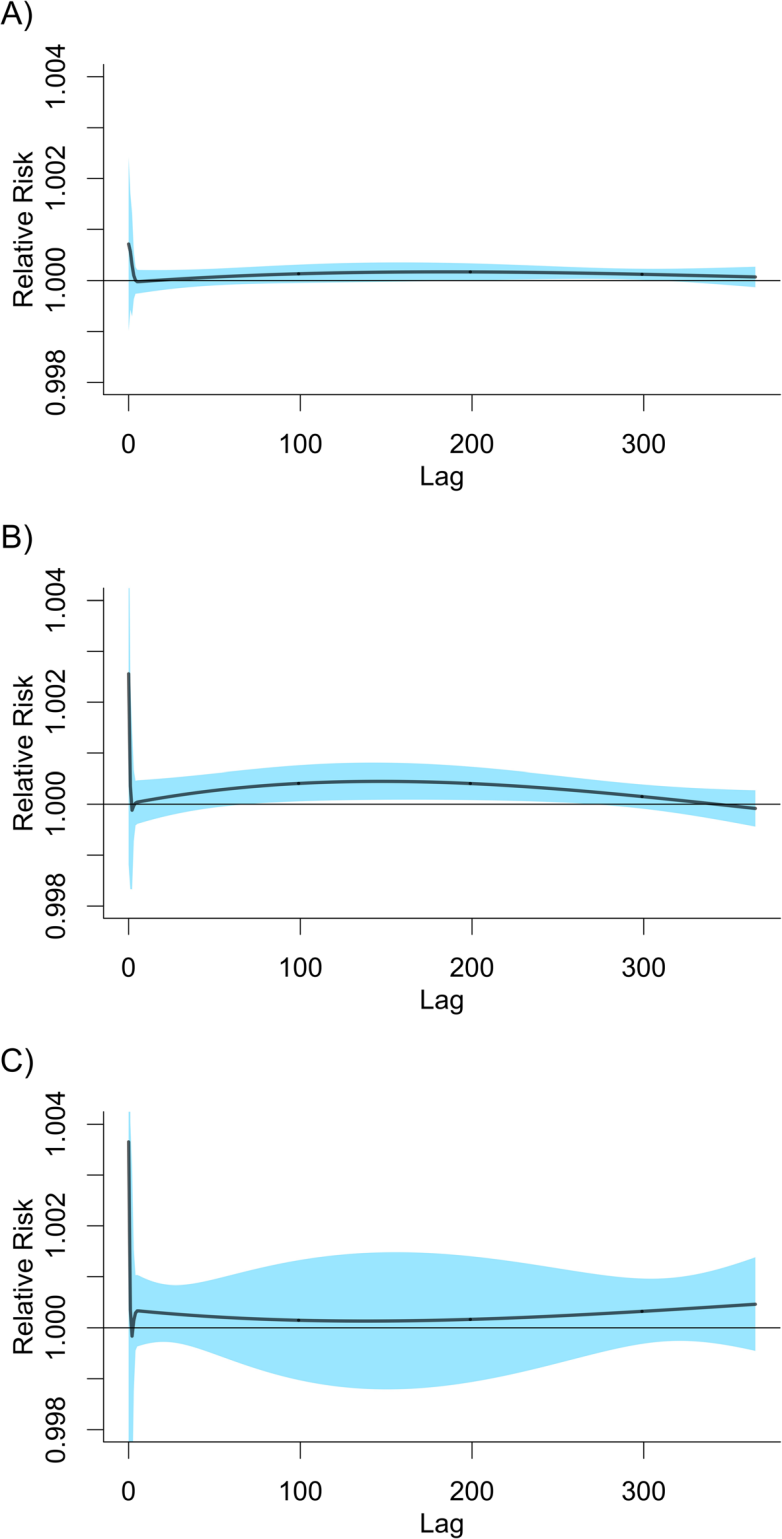
Estimated lag patterns of the association between a 10 μg/m^3^ increase of PM_10_ and mortality in seven major cities of South Korea, 2006–2013. **a** All-cause mortality. **b** Cardiovascular mortality. **c** Respiratory mortality

Figure 4 presents the percentage increase in all-cause mortality risk, cardiovascular mortality risk, and respiratory mortality risk by exposure duration. These increasing patterns reconcile previous observations that the association between exposure to PM and mortality increases with exposure duration: roughly similar to 5–15% increase of mortality per 20 μg/m^3^ increase of one-year exposure to PM_10_ [32, 33]. Figure 5 presents corresponding lag patterns.

## Discussion

We addressed a challenging issue in estimating the effect of long-term exposure on health outcomes using time-series analysis – adjustment for seasonality and long-term time-trend that do not mask the effect and not reduce substantially variability of residuals of exposure series. NCS(*t*,*p*df/year) that is frequently used in time-series studies may lead to very high standard errors, which makes it difficult to infer the magnitude of the association between long-term exposure and health outcomes. Our results suggest that a combination of functions can be used to sufficiently adjust for seasonality and long-term time-trend and also allow for enough variability of residuals of exposure series, so that inflated standard errors can be avoided. We also showed that confounding by long-term exposure effects may arise in time-series studies for estimating short-term exposure effects; however, we postulate that this confounding might not be an issue for air pollution time-series studies because adjustment methods for seasonality and long-term time-trend could adjust for this confounding.

The effect of long-term exposure may be more likely to be confounded by seasonality and time-trend than that of short-term exposure. Higher df of NCS would provide stricter adjustment but reduce variability of residuals of exposure series. A combination of functions allows for higher variability of residuals of exposure series but may require additional adjustments for seasonality, depending on data applied, what covariates are measured and how they are adjusted. For example, deviations of seasonality in an outcome series may still exist even after distributed lags of environmental variables that has strong seasonality such as temperature [38] are adjusted. Other examples may include seasonality in an outcome series attributable to influenza epidemic and longer-term exposure than exposure of interest. Therefore, sensitivity analyses are recommended to determine sufficient but not redundant adjustment for seasonality, time-trend, and possible confounding by longer-term exposure as we did in our real-data analyses.

In line with the merits of sensitivity analyses, overfitting must be considered as it increases variance estimates and can yield highly variable estimates of an association. Sometimes, to our experience, statistically significant but spurious associations such as lag patterns of NCS (4 or 10df/year) in Fig. 3 may come out. To avoid overfitting, identification of residual variance of exposure series may be helpful, as in Table 1. Comparing lag patterns estimated across different lag models may also guide whether overfitting arises or not because highly variable estimates would come out from different lag models when standard errors of coefficient estimates of interest are inflated [13].

Some limitations are noted. We did not extend our focus into longer-term exposure beyond 1 year in real-data analysis. We found that functions of variables representing time explain to a larger extent the variability of longer-term fluctuation (e.g., 2 years) in an exposure series. Thus, there would be smaller variability of residuals of exposure series. This issue may be overcome with collecting more informative data. Second, we did not address exposure misclassification theoretically and analytically. So, we cannot rule out the possibility that exposure misclassification might affect exposure durations to different degrees (such as Figs. 4 and 5), but the overall impact on an estimate of the cumulative coefficient $\overline{\beta}$ might be toward the null [7, 8].

Nevertheless, an estimate affected by exposure misclassification in the context of personal exposure may be seen as an estimate of the effect of ambient air pollution in a given region that is of public health importance. For example, time-series analysis could be used to address cessation lag [39] of ambient air pollution exposures in a given population, which is a critical issue in understanding efficiency of ambient air pollution interventions. Changes of loss of life expectancy in that region as a measure of the severity of public health burden [40, 41] may also be estimated considering long-term exposure effect without cohort data [23].

## Conclusions

We demonstrated that long-term exposure effects can be estimated using time-series analysis by addressing confounding by seasonality and long-term time-trend, while maintaining efficiency. Since maintaining efficiency may lead to less strict adjustment for seasonality and long-term time-trend, sensitivity analyses should be conducted to confirm that adjustment is sufficiently made. Rich case-only data such as death certificates and hospitalization records combined with repeated measurements of environmental determinants across countries would have high potentials for investigating the effects of long-term exposure on health outcomes allowing for unique contexts of local populations.

## Supplementary Information

**Additional file 1.** Simulation methods and additional results of simulations. **Figure S1.** Two hypothetical observable lag patterns of the association between 1 μg/m^3^ increase of exposure to PM_10_ and mortality, used to generate simulation samples (the last row of panels). A) and B) Hypothetical actual effect (identical). C) Mortality displacement for a few days to several weeks for Observable lag pattern 1. D) Mortality displacement for a few days to several weeks for Observable lag pattern 2. E) Mortality displacement for few days to two years for Observable lag pattern 1. F) Mortality displacement for few days to two years for Observable lag pattern 2. G) Observable lag patterns 1. H) Observable lag pattern 2. **Figure S2.** An example of simulated samples. A) all-cause death series. B) PM_10_ series. **Table S1.** Bias and Standard Deviation for the Association between a Two-Day Moving Average of PM_10_ (lag0–1) and All-Cause Mortality Estimated by a Model with Different Adjustment Methods for Seasonality and Long-Term Time-Trend over 5000 Samples

**Additional file 2.** Real data analysis methods and additional results. **Table S2.** Percentage increase and 95% confidence intervals of cardiovascular mortality risk per a 10 μg/m^3^ increase in two-day moving average of PM_10_ (lag0–1) or one-year distributed lags of PM_10_ (lag0–365) in seven major cities of South Korea, 2006–2013**. Table S3.** Percentage increase and 95% confidence intervals of respiratory mortality risk per a 10 μg/m^3^ increase in two-day moving average of PM_10_ (lag0–1) or one-year distributed lags of PM_10_ (lag0–365) in seven major cities of South Korea, 2006–2013**. Figure S3.** Time-series of logarithm of daily all-cause deaths predicted by models with two different adjustments for seasonality and long-term time trend and their absolute differences. A) Time-series in Seoul, 2006–2013 B) Absolute differences in Seoul. C) Time-series in Daegu, 2006–2013. D) Absolute differences in Daegu. Grey vertical dotted lines in B) and D) denote 80th, 90th, and 98th percentile of absolute differences. **Figure S4.** Results of additional sensitivity analyses in seven major cities of South Korea, 2006–2013. A) All-cause mortality. B) Cardiovascular mortality. C) Respiratory mortality. Percentage increases estimated by models with NCS(doy, 20df)+I(year)+NCS(week, 3df)+NCS(month, 3df) in Table 3 (for all-cause mortality), Table S2 (for cardiovascular mortality), and Table S3 (for respiratory mortality) were duplicated for comparison (red triangles).

## Data Availability

Daily number of deaths we used are not available without permission of Statistics Korea. City-level mortality data are publicly downloadable at the “Download service” section in MicroData Integrated Service (MDIS) by Statistics Korea (https://mdis.kostat.go.kr/index.do, in Korean) and more detailed mortality data can be purchased at the “Use Microdata” section in MDIS. Data for air pollution are publicly downloadable at the “Finally confirmed data” section in Air Korea (https://www.airkorea.or.kr/web, in Korean). Data for meteorological factors are publicly downloadable at the “Data” section in Data Portal by Korean Metrological Administration (https://data.kma.go.kr/, in Korean). R codes for simulations and real-data analysis are provided in the first author’s website, http://hkimresearch.com, or Github, https://github.com/HonghyokKim/AlternativeAdjustment.

## Abbreviations

PM_10_: Particulate matter with aerodynamic diameter ≤10 μm
NCS: Natural cubic spline
df: Degrees of freedom
NCS(*t*,*p*df/year): NCS of time (*t*) throughout time-series (or the study period) with *p*df per year
I(year): An indicator function of the order of year throughout time-series (or the study period)
doy: Day of the year
NCS(doy,*p*df): NCS of doy with *p*df
NCS(week,*p*df): NCS of the order of week throughout time-series (or the study period) with *p*df
NCS(month,*p*df): NCS of the order of month throughout time-series (or the study period) with *p*df
SD: Standard deviation
