# Supplementary material for: Alternative adjustment for seasonality and long-term time-trend in time-series analysis for long-term environmental exposures and disease counts
Source: BMC Med Res Methodol. 2021 Jan 4;21:2. doi: 10.1186/s12874-020-01199-1 (PMC7780665; doi:10.1186/s12874-020-01199-1)
Supplement: Supplementary file 1 — Additional file 1. Simulation methods and additional results of simulations. Figure S1. Two hypothetical observable lag patterns of the association between 1 μg/m3 increase of exposure to PM10 and mortality, used to generate simulation samples (the last row of panels). A) and B) Hypothetical actual effect (identical). C) Mortality displacement for a few days to several weeks for Observable lag pattern 1. D) Mortality displacement for a few days to several weeks for Observable lag pattern 2. E) Mortality displacement for few days to two years for Observable lag pattern 1. F) Mortality displacement for few days to two years for Observable lag pattern 2. G) Observable lag patterns 1. H) Observable lag pattern 2. Figure S2. An example of simulated samples. A) all-cause death series. B) PM10 series. Table S1. Bias and Standard Deviation for the Association between a Two-Day Moving Average of PM10 (lag0–1) and All-Cause Mortality Estimated by a Model with Different Adjustment Methods for Seasonality and Long-Term Time-Trend over 5000 Samples [file 12874_2020_1199_MOESM1_ESM.docx]

**Additional File 1. Simulation methods and additional results of simulations**

***Table and Figure List***

**Figure S1.** Two hypothetical observable lag patterns of the association between 1μg/m^3^ increase of exposure to PM_10_ and mortality, used to generate simulation samples (the last row of panels). A) and B) Hypothetical actual effect (identical). C) Mortality displacement for a few days to several weeks for Observable lag pattern 1. D) Mortality displacement for a few days to several weeks for Observable lag pattern 2. E) Mortality displacement for few days to two years for Observable lag pattern 1. F) Mortality displacement for few days to two years for Observable lag pattern 2. G) Observable lag patterns 1. H) Observable lag pattern 2.

**Figure S2.** An example of simulated samples. A) all-cause death series. B) PM_10_ series.

**Table S1.** Bias and Standard Deviation for the Association between a Two-Day Moving Average of PM_10_ (lag0–1) and All-Cause Mortality Estimated by a Model with Different Adjustment Methods for Seasonality and Long-Term Time-Trend over 5,000 Samples

We introduce steps to generate simulation samples below. All analysis was conducted with R software 3.5.3 (R Foundation for Statistical Computing)

***PM_10_ time-series***

1. Regress time-series of the logarithm of PM_10_ in Seoul, South Korea from 2002 to 2013 on the following variables
   1. NCS^*^(day of the week, 10df) + I^**^(year) + NCS(month, 5df) + NCS(week, 5df)
   2. Indicator of national holiday
   3. Indicator of day of the week

^*^NCS(*variable name*, *x*df) denotes a natural cubic spline of *variable name* with *x* degrees of freedom

^**^I denotes an indicator function

1. Extract 1-day lagged residuals and 2-day lagged residuals from the linear regression model
2. Regress the logarithm of PM_10_ time-series in Seoul from 2002 to 2013 on the following variables
   1. NCS(day of the week, 10df) + I(year) + NCS(month, 5df) + NCS(week, 5df)
   2. Indicator of national holiday
   3. Indicator of day of the week
   4. Cross-basis for temperature: One basis for temperature from lag0 to lag21 constrained by a cubic B-spline with internal knots on the logarithm scale defined by 7 degrees of freedom (df). The other basis for non-linear association between temperature and PM_10_, specified by a quadratic B-spline with internal knots of 10^th^, 50^th^, and 90^th^ percentile of temperature.
   5. NCS(relative humidity, 3df)
   6. Indicator of influenza epidemic
   7. 1-day lagged residuals
   8. 2-day lagged residuals
3. Extract predicted values from the OLS regression model, and also extract the standard deviation estimate of residuals from this model
4. Generate time-series of the logarithm of PM_10_ using the normal distribution with the predicted values (as a mean) and the standard deviation estimate of residuals (as a standard deviation)^*^

^*^For high concurvity, divide the standard deviation estimate by 10.

1. Exponentiate generated time-series of the logarithm of PM_10_
2. Repeat 5 and 6 to generate 5,000 samples

***All-cause mortality series***

1. Regress time-series of all-cause mortality cases in Seoul from 2002 to 2013 on the following variables using a generalized linear model with Quasi-Poisson distribution (log-link)
   1. NCS(day of the week, 10df) + I(year) + NCS(month, 5df) + NCS(week, 5df)
   2. Indicator of national holiday
   3. Indicator of day of the week
   4. Cross-basis for temperature: One basis for temperature from lag0 to lag21 constrained by a cubic B-spline with internal knots on the logarithm scale defined by 7df. The other basis for non-linear association between temperature and mortality, specified by a quadratic B-spline with internal knots of 10^th^, 50^th^, and 90^th^ percentile of temperature.
   5. NCS(relative humidity, 3df)
   6. Indicator of influenza epidemic
2. Extract predicted values of the logarithm of mortality series from the Poisson regression model.
3. Calculate a product of a lag pattern (Figure 2, Figure S1) and distributed lags (lag0 to lag730) of a generated PM_10_ time-series
4. Sum 2 and 3 and exponentiate it.
5. Generate time-series of all-cause mortality cases using the Poisson distribution with the mean of the calculated values from 4.
6. Repeat 5 to generate 5,000 samples for each setting with respect to lag patterns, concurvity of PM_10_ time-series


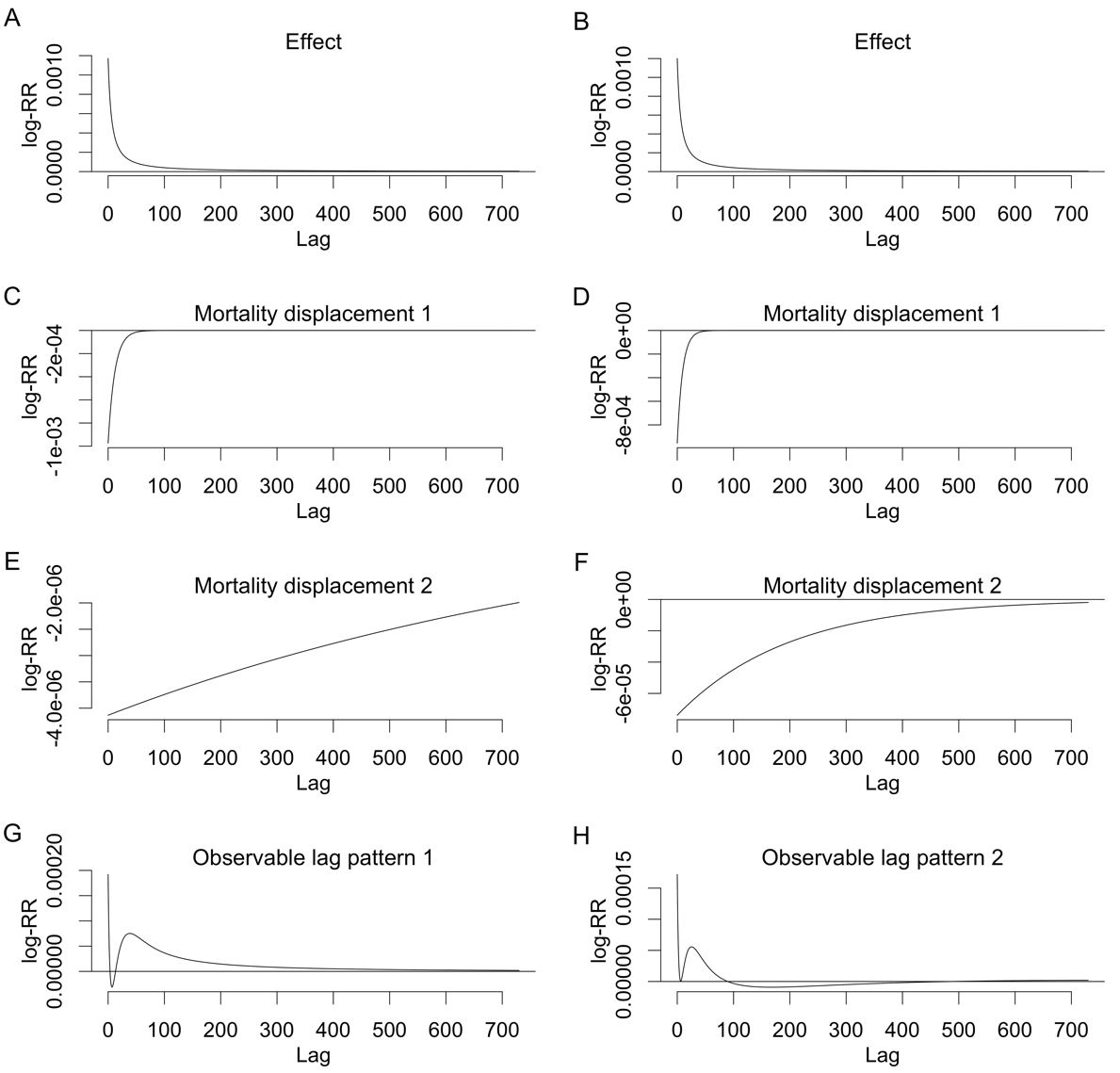


**Figure S1.** Two hypothetical observable lag patterns of the association between 1μg/m^3^ increase of exposure to PM_10_ and mortality, used to generate simulation samples (the last row of panels). A) and B) Hypothetical actual effect (identical). C) Mortality displacement for a few days to several weeks for Observable lag pattern 1. D) Mortality displacement for a few days to several weeks for Observable lag pattern 2. E) Mortality displacement for few days to two years for Observable lag pattern 1. F) Mortality displacement for few days to two years for Observable lag pattern 2. G) Observable lag patterns 1. H) Observable lag pattern 2.

Abbreviation: RR, relative risk.


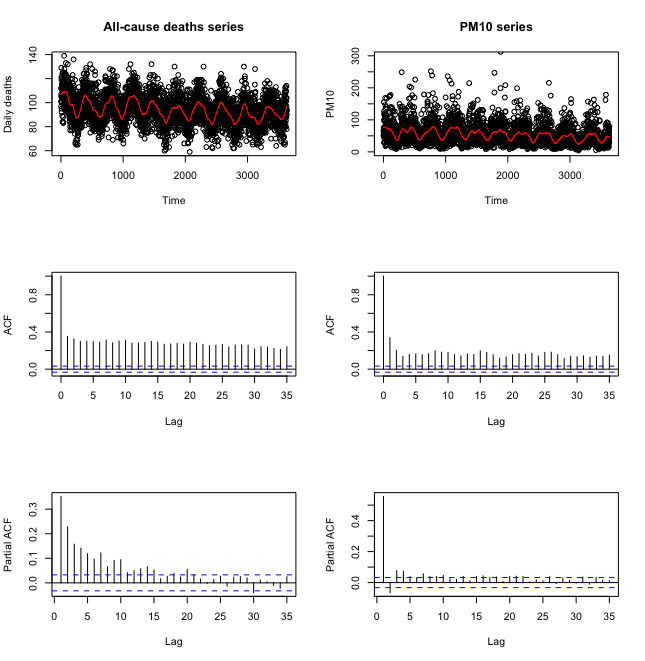


**Figure S2.** An example of simulated samples. A) all-cause death series. B) PM_10_ series. Abbreviation: ACF, autocorrelation function.

***Estimating association between short-term and long-term exposure to PM_10_ and all-cause mortality***

To estimate associations between short-term and long-term exposure to PM_10_ and all-cause mortality for generated samples, we fitted a generalized linear model with Quasi-Poisson distribution including the following variables:

- 1. PM_10_ variables: for short-term exposure, two-day moving average of PM_10_ (lag0 to lag1); for long-term exposure, distributed lags of PM_10_ from lag0 to lag730 constrained by NCS with internal knots on the logarithm scale defined by 10df.
  2. (For short-term only) distributed lags of PM_10_ from lag2 to lag730 constrained by a NCS with internal knots on the logarithm scale defined by 10df.
  3. NCS(day of the week, 10df) + I(year) + NCS(month, 5df) + NCS(week, 5df)
  4. Indicator of national holiday
  5. Indicator of day of the week
  6. Cross-basis for temperature: One basis for temperature from lag0 to lag21 constrained by a cubic B-spline with internal knots on the logarithm scale defined by 7df. The other basis for non-linear association between temperature and mortality, specified by a quadratic B-spline with internal knots of 10^th^, 50^th^, and 90^th^ percentile of temperature.
  7. A NCS(relative humidity, 3df)
  8. Indicator of influenza epidemic

**Table S1.** Bias and standard deviation for the association between a two-day moving average of PM_10_ (lag0–1) and all-cause mortality estimated by a model with different adjustment methods for seasonality and long-term time-trend over 5,000 Samples based on time-series data for Seoul, 2002–2013.

|  | **Observable Lag pattern 1** | | | **Observable Lag Pattern 2** | | |
| --- | --- | --- | --- | --- | --- | --- |
| **Adjustment** | **Bias (%)** | **SD^a^** | **Coverage^b^**  **(%)** | **Bias (%)** | **SD^a^** | **Coverage^b^**  **(%)** |
| Not adjusted | 147.7 | 0.0010 | 0.2 | -128.9 | 0.0009 | 1.0 |
| NCS(*t*,4df/year) | -14.7 | 0.0009 | 90.6 | -11.7 | 0.0008 | 90.6 |
| NCS(*t*,5df/year) | -13.2 | 0.0009 | 90.9 | -14.0 | 0.0009 | 90.9 |
| NCS(*t*,6df/year) | -10.5 | 0.0009 | 92.3 | -14.4 | 0.0009 | 92.3 |
| NCS(*t*,7df/year) | -5.4 | 0.0009 | 93.7 | -12.5 | 0.0009 | 93.7 |
| NCS(*t*,8df/year) | -2.1 | 0.0009 | 94.4 | -11.7 | 0.0009 | 94.4 |
| NCS(*t*,9df/year) | 0.5 | 0.0009 | 94.7 | -10.3 | 0.0009 | 94.7 |
| NCS(*t*,10df/year) | 0.4 | 0.0009 | 95.0 | -11.3 | 0.0009 | 95.0 |
| NCS(*t*,11df/year) | 2.8 | 0.0009 | 95.0 | -9.6 | 0.0009 | 95.0 |
| NCS(*t*,12df/year) | 2.7 | 0.0009 | 94.9 | -9.7 | 0.0009 | 94.9 |
| NCS(*t*,13df/year) | 4.0 | 0.0009 | 94.9 | -8.6 | 0.0009 | 94.9 |
| NCS(*t*,14df/year) | 5.1 | 0.0009 | 94.8 | -7.7 | 0.0009 | 94.8 |
| NCS(*t*,15df/year) | 5.8 | 0.0009 | 94.7 | -6.5 | 0.0009 | 94.7 |
| NCS(*t*,20df/year) | 8.4 | 0.0009 | 94.4 | -2.6 | 0.0009 | 94.4 |
| Not adjusted &  lag2–730 adjusted | -3.2 | 0.0008 | 93.5 | -3.0 | 0.0008 | 93.5 |
| NCS(*t*,10df/year) &  lag2–730 adjusted | 1.1 | 0.0010 | 95.0 | 2.8 | 0.0010 | 95 |
| NCS(*t*,20df/year) &  lag2–730 adjusted | -4.4 | 0.0012 | 94.2 | -1.5 | 0.0012 | 94.2 |

Abbreviation: df, degrees of freedom; NCS, natural cubic spline; NCS(*t*,*p*df/year), NCS of time throughout the study period with *p*df per year; PM_10_, particulate matter with aerodynamic diameter ≤10µm; SD, standard deviation.

^a^SD of estimates of the overall cumulative coefficient (as 10 μg/m^3^ increase of PM_10_)

^b^Nominal coverage of 95% confidence intervals.
