# Supplementary material for: Alternative adjustment for seasonality and long-term time-trend in time-series analysis for long-term environmental exposures and disease counts
Source: BMC Med Res Methodol. 2021 Jan 4;21:2. doi: 10.1186/s12874-020-01199-1 (PMC7780665; doi:10.1186/s12874-020-01199-1)
Supplement: Supplementary file 2 — Additional file 2. Real data analysis methods and additional results. Table S2. Percentage increase and 95% confidence intervals of cardiovascular mortality risk per a 10 μg/m3 increase in two-day moving average of PM10 (lag0–1) or one-year distributed lags of PM10 (lag0–365) in seven major cities of South Korea, 2006–2013. Table S3. Percentage increase and 95% confidence intervals of respiratory mortality risk per a 10 μg/m3 increase in two-day moving average of PM10 (lag0–1) or one-year distributed lags of PM10 (lag0–365) in seven major cities of South Korea, 2006–2013. Figure S3. Time-series of logarithm of daily all-cause deaths predicted by models with two different adjustments for seasonality and long-term time trend and their absolute differences. A) Time-series in Seoul, 2006–2013 B) Absolute differences in Seoul. C) Time-series in Daegu, 2006–2013. D) Absolute differences in Daegu. Grey vertical dotted lines in B) and D) denote 80th, 90th, and 98th percentile of absolute differences. Figure S4. Results of additional sensitivity analyses in seven major cities of South Korea, 2006–2013. A) All-cause mortality. B) Cardiovascular mortality. C) Respiratory mortality. Percentage increases estimated by models with NCS(doy, 20df)+I(year)+NCS(week, 3df)+NCS(month, 3df) in Table 3 (for all-cause mortality), Table S2 (for cardiovascular mortality), and Table S3 (for respiratory mortality) were duplicated for comparison (red triangles). [file 12874_2020_1199_MOESM2_ESM.docx]

**Additional File 2. Real data analysis methods and additional results**

***Table and Figure List***

**Table S2.** Percentage increase and 95% confidence intervals of cardiovascular mortality risk per a 10µg/m^3^ increase in two-day moving average of PM_10_ (lag0–1) or one-year distributed lags of PM_10_ (lag0–365) in seven major cities of South Korea, 2006–2013

**Table S3.** Percentage increase and 95% confidence intervals of respiratory mortality risk per a 10µg/m^3^ increase in two-day moving average of PM_10_ (lag0–1) or one-year distributed lags of PM_10_ (lag0–365) in seven major cities of South Korea, 2006–2013

**Figure S3.** Time-series of logarithm of daily all-cause deaths predicted by models with two different adjustments for seasonality and long-term time trend and their absolute differences. A) Time-series in Seoul, 2006–2013 B) Absolute differences in Seoul. C) Time-series in Daegu, 2006–2013. D) Absolute differences in Daegu. Grey vertical dotted lines in B) and D) denote 80^th^, 90^th^, and 98^th^ percentile of absolute differences.

**Figure S4.** Results of additional sensitivity analyses in seven major cities of South Korea, 2006–2013. A) All-cause mortality. B) Cardiovascular mortality. C) Respiratory mortality. Percentage increases estimated by models with NCS(doy, 20df)+I(year)+NCS(week, 3df)+NCS(month, 3df) in Table 3 (for all-cause mortality), Table S2 (for cardiovascular mortality), and Table S3 (for respiratory mortality) were duplicated for comparison (red triangles).

Time-series datasets for seven major cities of South Korea (Seoul, Busan, Daegu, Incheon, Gwangju, Daejeon, and Ulsan) from 2006 to 2013 were used in this study. Mortality data were obtained from Statistics Korea. All-cause mortality (ICD-10^th^ revision: A00-R99), cardiovascular mortality (I00-99), and respiratory mortality (J00-99) were identified, and daily number of death cases were calculated. A total of all-cause mortality, cardiovascular mortality and respiratory mortality were 701,266 cases, 181,376 cases, 51,534 cases, respectively. Hourly measurements for PM_10_ and O_3_ at one monitoring station per district (i.e., administrative unit lower than the metropolitan city) were obtained from the National Institute of Environmental Research. City-specific 1-hour concentrations of PM_10_ and O_3_ were calculated, and then 24-hour average of PM_10_ and maximum of 8-hour moving average of O_3_ were calculated. We treated values calculated from >25% missing values as missing. There were few missing values, and we imputed an annual average. The average of city-specific PM_10_ concentration ranged from 44.5 μg/m^3^ in Daejeon to 57.0 μg/m^3^ in Incheon. For temperature and relative humidity, hourly measurements at the city center obtained from the Korean Meteorological Administration were averaged to daily values. There were no missing values for temperature and relative humidity. Influenza epidemic was based on the number of hospital visits for influenza, which was obtained from the National Health Insurance System.

To estimate the effect of short-term and long-term exposure to PM_10_ on mortality, we fitted a generalized linear model with QuasiPoisson distribution including the following variables:

1. PM_10_ variables: for short-term exposure, two-day moving average of PM_10_ (lag0 to lag1); for long-term exposure, distributed lags of PM_10_ from lag0 to lag365 constrained by NCS with internal knots (i.e., 0.9137158, 2.2694299, and 5.6366674)
2. Distributed lags of O_3_^*^ from lag0 to lag45 constrained by NCS with internal knots (i.e., 0.9137158, 2.2694299, and 5.6366674)

^*^Omission provided similar results.

1. NCS(day of the week, 10df) + I(year) + NCS(month, 5df) + NCS(week, 5df)
2. Indicator of national holiday
3. Indicator of day of the week
4. Cross-basis for temperature: One basis for temperature from lag0 to lag14 (or lag0 to lag21^*^) constrained by an NCS with internal knots (i.e., 0.8260537, 1.8548595, 4.1649883, and 9.3522593). The other basis for non-linear association between temperature and PM_10_, specified by a quadratic B-spline with internal knots of 10^th^, 50^th^, and 90^th^ percentile of temperature.

^*^This analysis provided consistent point estimates with wider confidence intervals than those in the main analysis presented in the main manuscript and Web Tables 2-3.

1. NCS(relative humidity, 3df)
2. Indicator of influenza epidemic

**Table S2.** Percentage increase and 95% confidence intervals of cardiovascular mortality risk per a 10µg/m^3^ increase in two-day moving average of PM_10_ (lag0–1) or one-year distributed lags of PM_10_ (lag0–365) in seven major cities of South Korea, 2006–2013

| **Adjustment method for seasonality and long-term time-trend^a^** | **Two-day moving average**  **(lag0–1)** | | | | **One-year distributed lags** | |
| --- | --- | --- | --- | --- | --- | --- |
|  | **lag2–365 not adjusted** | | **lag2–365 adjusted** | | **(lag0–365)** | |
|  | **PI** | **95% CI** | **PI** | **PI** | **95% CI** | **95% CI** |
| Not adjusted | 0.06 | -0.54, 0.67 | 0.25 | -0.07, 0.57 | -1.04 | -5.94, 4.12 |
| NCS(t, 7df/year) | 0.27 | -0.12, 0.66 | 0.26 | -0.28, 0.80 | -35.42 | -82.64, 140.26 |
| NCS(t, 10df/year) | 0.26 | -0.13, 0.65 | 0.27 | -0.26, 0.81 | -55.82 | -95.92, 378.14 |
| NCS(t, 12df/year) | 0.26 | -0.14, 0.66 | 0.30 | -0.31, 0.91 | -71.48 | -98.39, 403.8 |
| NCS(doy, 20df)+I(year) | 0.20 | -0.27, 0.68 | 0.36 | -0.10, 0.82 | 6.94 | -2.10, 16.81 |
| 10,3,3 | 0.19 | -0.26, 0.63 | 0.30 | -0.16, 0.76 | 10.39 | 0.29, 21.50 |
| 20,3,3 | 0.20 | -0.25, 0.66 | 0.32 | -0.13, 0.77 | 10.76 | 0.62, 21.93 |
| 30,3,3 | 0.26 | -0.18, 0.70 | 0.37 | -0.06, 0.81 | 10.88 | 0.56, 22.26 |
| 20,4,4 | 0.22 | -0.24, 0.67 | 0.32 | -0.12, 0.77 | 12.29 | 2.12, 23.48 |
| 20,5,5 | 0.19 | -0.26, 0.65 | 0.28 | -0.19, 0.76 | 10.87 | -0.55, 23.61 |
| 20,6,6 | 0.15 | -0.29, 0.60 | 0.25 | -0.22, 0.73 | 6.41 | -3.50, 17.34 |
| 20,10,10 | 0.15 | -0.31, 0.60 | 0.23 | -0.24, 0.70 | -3.81 | -22.43, 19.28 |
| 20,15,15 | 0.17 | -0.29, 0.62 | 0.28 | -0.18, 0.74 | -8.84 | -38.52, 35.17 |

Abbreviation: CI, confidence interval; df, degrees of freedom; doy, day of the year; I(year), an indicator function of year throughout the study period; NCS, natural cubic spline; NCS(doy,*p*df), NCS of doy with *p*df; NCS(t,*p*df/year), NCS of time throughout the study period with *p*df per year; PI, percentage increase; PM_10_, particulate matter with aerodynamic diameter ≤10µm.

^a^NCS(doy,*p*df)+I(year)+NCS(week,*q*df)+NCS(month,*r*df) is described as *p*,*q*,*r*; NCS(week,*q*df) is NCS of the order of week throughout the study period with *q*df; NCS(month,*r*df) is NCS of the order of month throughout the study period with *r*df.

**Table S3.** Percentage increase and 95% confidence intervals of respiratory mortality risk per a 10µg/m^3^ increase in two-day moving average of PM_10_ (lag0–1) or one-year distributed lags of PM_10_ (lag0–365) in seven major cities of South Korea, 2006–2013

| **Adjustment method for seasonality and long-term time-trend^a^** | **Two-day moving average**  **(lag0–1)** | | | | **One-year distributed lags** | |
| --- | --- | --- | --- | --- | --- | --- |
|  | **lag2–365 not adjusted** | | **lag2–365 adjusted** | | **(lag0–365)** | |
|  | **PI** | **95% CI** | **PI** | **95% CI** | **PI** | **95% CI** |
| Not adjusted | -2.08 | -2.63, -1.53 | 0.10 | -0.57, 0.77 | -31.39 | -35.25, -27.30 |
| NCS(t, 7df/year) | 0.62 | 0.05, 1.20 | 0.57 | -0.29, 1.42 | 70.55 | -90.16, 2857.43 |
| NCS(t, 10df/year) | 0.55 | -0.04, 1.14 | 0.25 | -0.83, 1.33 | 7.88 | -99.44, 20860.37 |
| NCS(t, 12df/year) | 0.58 | -0.01, 1.17 | 0.21 | -0.87, 1.31 | -81.83 | -99.93, 4608.13 |
| NCS(doy, 20df)+I(year) | 0.58 | 0.02, 1.16 | 0.53 | -0.07, 1.14 | -1.21 | -25.35, 30.74 |
| 10,3,3 | 0.38 | -0.19, 0.95 | 0.42 | -0.18, 1.03 | 9.17 | -17.5, 44.45 |
| 20,3,3 | 0.38 | -0.19, 0.96 | 0.44 | -0.17, 1.06 | 9.34 | -17.77, 45.40 |
| 30,3,3 | 0.37 | -0.21, 0.95 | 0.42 | -0.19, 1.04 | 9.23 | -18.44, 46.28 |
| 20,4,4 | 0.40 | -0.18, 0.97 | 0.43 | -0.18, 1.05 | 12.17 | -12.7, 44.13 |
| 20,5,5 | 0.36 | -0.21, 0.94 | 0.37 | -0.24, 0.98 | 15.26 | -12.84, 52.43 |
| 20,6,6 | 0.30 | -0.28, 0.87 | 0.35 | -0.26, 0.96 | 6.32 | -19.81, 40.95 |
| 20,10,10 | 0.28 | -0.30, 0.86 | 0.35 | -0.27, 0.97 | 3.59 | -31.17, 55.91 |
| 20,15,15 | 0.37 | -0.21, 0.95 | 0.52 | -0.11, 1.15 | -18.55 | -69.78, 119.52 |

Abbreviation: CI, confidence interval; df, degrees of freedom; doy, day of the year; I(year), an indicator function of year throughout the study period; NCS, natural cubic spline; NCS(doy,*p*df), NCS of doy with *p*df; NCS(t,*p*df/year), NCS of time throughout the study period with *p*df per year; PI, percentage increase; PM_10_, particulate matter with aerodynamic diameter ≤10µm.

^a^NCS(doy,*p*df)+I(year)+NCS(week,*q*df)+NCS(month,*r*df) is described as *p*,*q*,*r*; NCS(week,*q*df) is NCS of the order of week throughout the study period with *q*df; NCS(month,*r*df) is NCS of the order of month throughout the study period with *r*df.

**
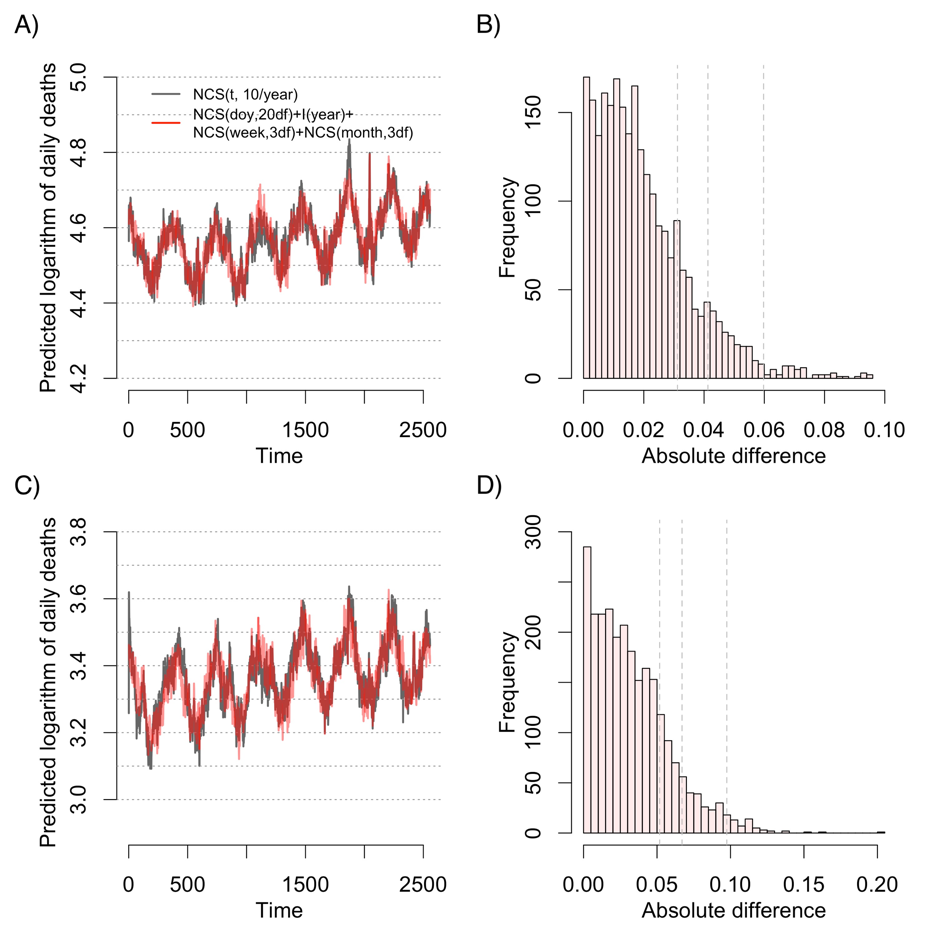
**

**Figure S3**. Time-series of logarithm of daily all-cause deaths predicted by models with two different adjustments for seasonality and long-term time trend and their absolute differences. A) Time-series in Seoul, 2006–2013. B) Absolute differences in Seoul. C) Time-series in Daegu, 2006–2013. D) Absolute differences in Daegu. Grey vertical dotted lines in B) and D) denote 80^th^, 90^th^, and 98^th^ percentile of absolute differences.


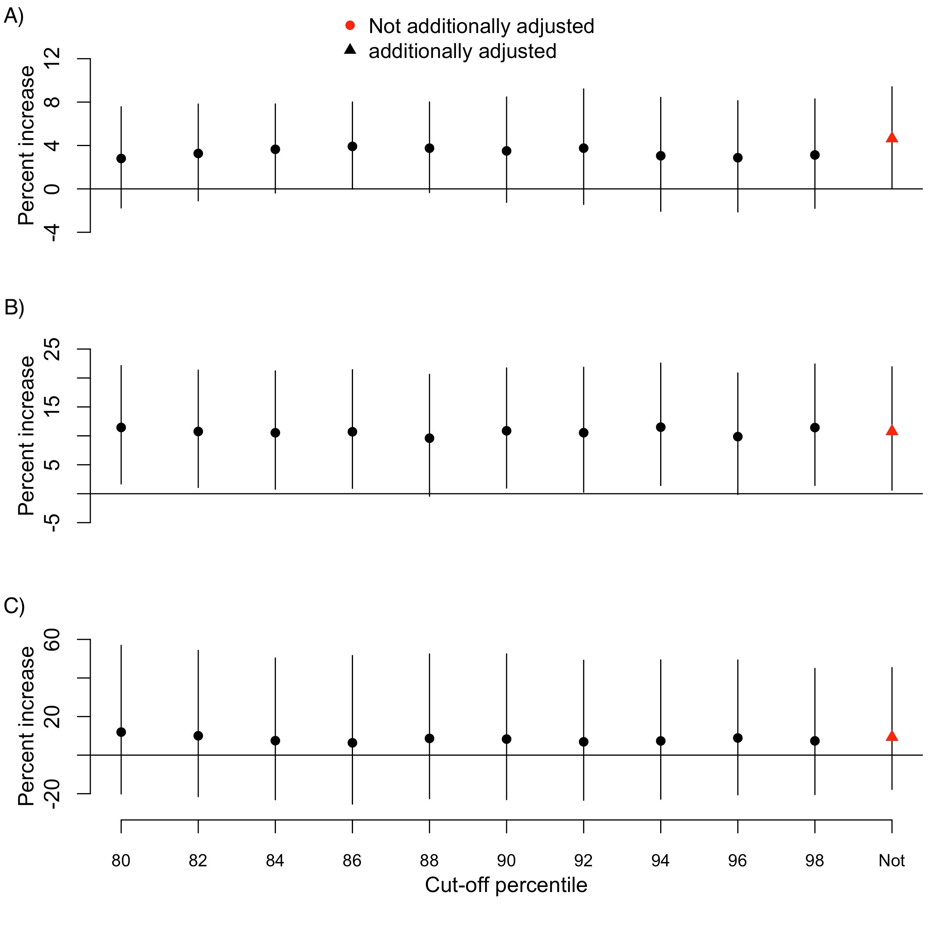


**Figure S4**. Results of additional sensitivity analyses in seven major cities of South Korea, 2006–2013. A) All-cause mortality. B) Cardiovascular mortality. C) Respiratory mortality. Percentage increases estimated by models with NCS(doy, 20df)+I(year)+NCS(week, 3df)+NCS(month, 3df) in Table 3 (for all-cause mortality), Table S2 (for cardiovascular mortality), and Table S3 (for respiratory mortality) were duplicated for comparison (red triangles).
